# Ureteral reimplantation during augmentation cystoplasty is not needed for vesicoureteral reflux in patients with neurogenic bladder: a long-term retrospective study

**DOI:** 10.1186/s12894-022-00997-7

**Published:** 2022-03-29

**Authors:** Hiroki Chiba, Takeya Kitta, Madoka Higuchi, Naohisa Kusakabe, Masafumi Kon, Michiko Nakamura, Nobuo Shinohara

**Affiliations:** Department of Renal and Genitourinary Surgery, Graduate School of Medicine, Hokkaido University, North-15 West-7 Kita-Ku, Sapporo, 060-8638 Japan

**Keywords:** Neurogenic bladder, Augmentation cystoplasty, Ureteral reimplantation, Vesicoureteral reflux

## Abstract

**Background:**

To investigate the need for ureteral reimplantation for vesicoureteral reflux (VUR) during augmentation cystoplasty (AC) in the long term.

**Methods:**

A total of 19 patients with a median age at surgery of 14 years (3–38 years) who underwent AC for neurogenic bladder with VUR between 1983 and 2016 were included in this study. The changes in VUR grade and urodynamic findings were retrospectively evaluated. We evaluated the renal function by periodic inspection of serum creatinine level and estimated glomerular filtration rate; eGFR.

**Results:**

The median follow-up period from AC was 14.8 years (5.7–30 years). VUR was detected in 19 patients, involving 27 ureters. Reflux grade was V in 6, IV in 9, III in 5, II in 6, and I in 1. Ureteral reimplantation was not performed in 18 patients (26 ureters), whereas it was done for 1 patient (1 ureter) in the early era of our experience. Postoperative videourodynamics showed that the reflux was radiologically not verifiable in 23 ureters (85%), was downgraded in 3 ureters (11%), and was unchanged in 1 ureter (3%). There were no cases of deterioration of VUR.

**Conclusions:**

Ureteral reimplantation is not necessary for VUR during augmentation cystoplasty.

## Background

Vesicoureteral reflux (VUR) occurs in patients with neurogenic bladder, which is a high-pressure and low-compliant bladder. High-grade VUR leads to recurrent urinary tract infections (UTIs), and thus causes severe renal failure. Initially, as conservative treatment, clean intermittent catheterization (CIC) and anticholinergic therapy were generally conducted. Intravesical injection of botulinum toxin A is also effective to improve bladder compliance and capacity. Endoscopic antireflux surgery may be useful when bladder capacity and compliance are near normal [1]. If these conservative managements are ineffective, augmentation cystoplasty (AC) is generally accepted as one of the standard therapeutic options. Although various bowel segments such as the sigmoid colon, cecum, and ileum have been used for AC historically, the most widely used segment for AC recently is the ileum [2]. If bowel is unusable, the stomach can be an alternative organ. Regarding the treatment of VUR in patients with neurogenic bladder, whether ureteral reimplantation should be simultaneously performed with AC for patients with VUR is controversial [3]. It is accepted that VUR in neurogenic bladder is predominantly a secondary reflux, which is caused by small bladder capacity, poor bladder compliance, and dysfunctional voiding [1]. We have not routinely performed ureteral reimplantation with AC because these refluxes could improve when a low-pressure system is created. The aim of this study was to evaluate the need for ureteral reimplantation with AC for patients with VUR.

## Methods

### Patients

This study included 19 patients (10 male, 9 female) who underwent AC for neurogenic bladder with VUR between March 1983 and March 2016 in our hospital. A minimum follow-up of 5 years after AC was necessary for inclusion in this study. All patients had been treated conservatively with CIC and anticholinergic agents. The indications for AC for neurogenic bladder were low bladder compliance, urinary incontinence, recurrent febrile UTIs (pyelonephritis, prostatitis, and epididymitis), high-grade VUR, and deterioration of renal function (CKD stage ≥ 3, or sequential deterioration of split renal function in renal scintigraphy), despite the conservative therapies. The changes in VUR grade, urodynamic findings, and postoperative complications were evaluated retrospectively. We evaluated the renal function by periodic inspection of serum creatinine level and estimated glomerular filtration rate; eGFR. The present study was approved by the Scientific Ethics Committee of Hokkaido University (# 020-0093).

### Surgical technique

AC was performed according to the techniques of Hautmann. The ileum has been our first choice of bowel segment for AC. The length of the ileum used for cystoplasty commonly depends on the body size of the patients, and at least 20 cm of the terminal ileum were preserved during dissection in all cases. The ileum segment was detubularized and formed into a “U” or “W” shape and then anastomosed to the opened original bladder. A urethral fascial sling was fashioned as a continence procedure in 5 patients (26%), and Malone’s antegrade continence enema (MACE) was performed to manage fecal incontinence in 7 patients (37%). Although ureteral reimplantation was not basically performed during AC in our institution, it was done for 1 early case (1 ureter) with high-grade VUR.

### Videourodynamics

Pre- and post-surgery videourodynamics was basically performed for all patients. The data of videourodynamics at more than 6 months after the surgery were used in this study. The methods of videourodynamics were according to the International Continence Society [4]. The Ellipse Urodynamic System (Andromeda, Taufkirchen/Potzham, Germany) was used for videourodynamics. Standard fluid cystometry was done with patients in a supine position, without sedation, using a 6-Fr double-lumen catheter and a 9-Fr rectal balloon catheter, with filling at a rate of less than 10% of the predicted bladder capacity per minute with warm saline. In accordance with the definition proposed by the International Continence Society, detrusor overactivity (DO) was defined as any involuntary detrusor contractions that occurred during the filling phase, which could be spontaneous or provoked, and which the patient could not completely suppress. Changes in bladder capacity and bladder compliance, the presence of DO, and the grade of VUR were evaluated.

### Statistical analysis

Statistical analysis was performed using Student’s *t*-test and the chi-squared test. A *P* value < 0.05 was considered significant. JMP pro 14.0 (SAS Institute) was used to analyze the data.

## Results

A total of 19 patients with a median age at surgery of 14 years (range 3–38 years) were included. Median follow-up from AC surgery was 14.8 years (range 5.7–30 years). The primary diagnoses causing neurogenic bladder were myelomeningocele, spinal cord lipoma, and imperforate anus (Table 1). Simultaneous procedures in the cystoplasty were fascial sling, antegrade continence enema, construction of a catheterizable channel, and ureteral reimplantation. Ureteral reimplantation was performed for only one patient in the very early stage of our experience. Perioperative complications are shown in Table 2. Postoperative complications included bladder stones in 8 patients (42%) and febrile UTIs in 6 patients (31%). Table 3 shows the changes in VUR grade from before to after surgery. VUR was found in 19 patients, involving 27 ureters. In a total of 27 ureters, reflux grade was V in 6, IV in 9, III in 5, II in 6, and I in 1. Postoperative videourodynamics showed that the reflux resolved in 23 ureters (85%), was downgraded in 3 ureters (11%), and was unchanged in 1 ureter (4%). There were no cases in which VUR deteriorated. On videourodynamics, the bladder capacity at which VUR occurred was significantly increased from 60 to 404 ml (*p* < 0.05), whereas detrusor pressure at the onset of VUR was not significantly different (Table 4). Regarding the renal function, eGFR and CKD stage was checked at the last visit. The median eGFR was 95 ml/min/1.73m^2^ (range 3.3–154) in 19 patients. Chronic kidney disease: CKD stage 1 in 11, stage 2 in 5, stage3 in 1, stage 4 in 0, stage5 in 2 patients were observed respectively. Chronic renal failure (CKD stage ≥ 3) developed in 3 patients (16%) during follow-up; 2 of 3 the patients developed stage 5 CKD and were started on renal replacement therapy. These 3 patients with renal failure have no VUR after the operation.

**Table 1:** Patients’ characteristics

	Number (%)
Number of patients	19
Male	10 (53)
Female	9 (47)
Age (y) (median, range)	14 (3–38)
Follow up (y) (median, range)	14.8 (5.7–30)
Diagnosis	
Myelomeningocele	12 (63)
Spinal lipoma	5 (26)
Anal atresia	2 (11)
Indications for AC	
Low-compliance bladder	7 (37)
Febrile UTI	6 (32)
High-grade VUR	6 (32)
Renal dysfunction	5 (26)
Surgical procedure with cystoplasty	
Fascial sling	5 (26)
Antegrade continence enema	7 (37)
Construction of catheterizable channel	2 (11)
Ureteral reimplantation	1 (5)

*AC* augmentation cystoplasty, *UTI* urinary tract infection, *VUR* vesicoureteral reflux

**Table 2:** Perioperative complications

	Number (%)
Early postoperative complication (≤ 30 days)	
Ileus	2 (11)
Febrile urinary tract infection	1 (5)
Surgical site infection	1 (5)
Late postoperative complication (> 30 days)	
Bladder stone	8 (42)
Febrile urinary tract infection	6 (31)
Metabolic acidosis	4 (21)
Folic acid deficiency	2 (11)
Perforation of enterocyctoplasty	1 (5)
Ileus	0 (0)

**Table 3:** Change in VUR grade from before to after surgery

VUR grade	Preoperative	Postoperative
0	0	23
I	1	2
II	6	2
III	5	0
IV	9	0
V	6	0

*VUR* vesicoureteral reflux

**Table 4:** Videourodynamic findings before and after augmentation cystoplasty

	Preoperative	Postoperative	*P* value
Bladder capacity (ml) (median, range)	200 (90–509)	400 (93–585)	*P* < 0.001
Bladder compliance (ml/cmH_2_O) (median, range)	7.5 (2.5–18.6)	28.1 (4.0–200)	*P* < 0.001
Detrusor overactivity (number, %)	9 (47)	2 (11)	NS
VUR (number, %)	19 (100)	4 (21)	*P* < 0.001
Bladder capacity at VUR occurred (ml) (median, range)	60 (10–300)	404 (50–500)	*P* < 0.05
Pdet when VUR occurred (cmH_2_O) (median, range)	6.5 (0–32)	8.5 (0–14)	NS

*VUR* vesicoureteral reflux

## Discussion

In management of VUR in neurogenic bladder, first of all, CIC with anticholinergics should be considered as conservative therapies. Injection of botulinum toxin A into the bladder is also one of effective conservative therapies. It is well known that these conservative therapies, which improve bladder capacity and compliance resolve VUR without surgery [1]. Considering the perioperative complications, and long-term management of augmented bladder, AC should be a last resort, therefore, indication of AC must be strictly determined.

This retrospective study investigated the change in VUR grade from before to after AC alone for patients with neurogenic bladder. The results showed that almost all cases of VUR were resolved or downgraded without ureteral reimplantation. Whether ureteral reimplantation should be done simultaneously with AC has been controversial. Because there have been very few prospective, randomized studies, there is no conclusive evidence about the usefulness of ureteral reimplantation with AC. Previous studies have shown that ureteral reimplantation should be performed in patients with low-pressure or high-grade VUR, ureterovesical junction obstruction, and severe upper urinary tract dilation [5–10]. Helmy et al. reported that unilateral high-grade VUR resolved in 9 of 11 (82%) cases, whereas bilateral high-grade VUR resolved in only 8 of 21 cases (38%) [6]. Moreover, UTI occurred in half of those patients during follow-up, so they recommended that antireflux surgery should be done for patients with bilateral high-grade VUR. Soygur et al. showed that VUR persisted in some patients, although AC was successful [8]. They found that VUR started at very low bladder pressure on preoperative videourodynamics. The authors hypothesized that primary ureterotrigonal insufficiency (short submucosal tunnel) may cause VUR, even if sufficient bladder capacity is secured, so that they concluded that such patients require ureteral reimplantation. Wang et al. also demonstrated that concomitant ureteral reimplantation should be performed for patients with low-bladder pressure VUR, high-grade VUR, or in those with concomitant ureterovesical junction (UVJ) stenosis [5].

In contrast, several reports demonstrated that routine ureteral reimplantation is not needed with AC [11–14]. Zang et al. argued that ureteral reimplantation is not necessary for all patients with VUR. In their study, 24/29 (83%) patients resolved completely, 3/29 (10%) improved, and 2/29 (7%) showed no change in reflux [11]. Their results are very similar to those of the current report. They stated that it is more important for prevention of UTI and renal failure to have safe bladder volume and to do CIC adequately than to perform reimplantation. Some other reports also demonstrated that ureter reimplantation was unnecessary with AC [12–15].

In the current study, of 27 ureters affected by reflux, 23 (85%) resolved and 3 (11%) were downgraded after AC without ureteral reimplantation. Although UTI occurred in 6 patients during the follow-up period after the AC procedure, VUR was resolved in 4 patients and downgraded in 2 of these patients. Moreover, renal failure has not occurred in these 6 patients during follow-up. Ureteric reimplantation may certainly repair VUR, but there are some disadvantages to doing this procedure with AC simultaneously, especially using the ileum. It is not easy to reimplant the ureter into a thick bladder wall, and the ureter is dilated in most cases, so that ureteral tailoring may be needed. There is a possibility that these complicated procedures result in prolonged operative time and increased surgical stress. In addition, we should also consider the risk of ureteral stricture as a postoperative complication. In terms of videourodynamics, bladder capacity at the onset of VUR was significantly increased after AC surgery, whereas detrusor pressure was unchanged. Although VUR occurred at low detrusor pressure after AC, the frequency of VUR was considered to be less than at pre-operation, because bladder capacity was greatly increased. It is important to perform CIC adequately to avoid the risk of VUR and UTI.

One of the important goals of AC is to protect upper urinary tract function. In the present study, 3 patients (16%) developed ≥ stage 3 CKD during follow-up, and 2 of the 3 patients started renal replacement therapy. However, there was no VUR in these patients after the operation. Husmann et al. reported that poor compliance with CIC could be a risk factor for chronic renal failure [16, 17]. In this regard, CIC through an abdominal stoma decreases the risk of chronic renal failure compared with CIC through the urethra in patients with obesity. Moreover, preoperative lower renal function at baseline may be related to renal function deterioration after surgery [2]. Thus, appropriate CIC after AC is important to prevent febrile UTI, and to protect upper urinary function, however, it is difficult to assess compliance of CIC in children and parents before the operation. Ureteral reimplantation may be beneficial for the patients with poor compliance. In addition, there may be a sub-population of children, those starting with poor renal function, that may actually benefit from reimplantation and perhaps delay to eventual complete renal failure.

## Conclusions

Although it is still controversial, from our study, we conclude that routine ureteral reimplantation is not necessary with AC in patients with VUR. This was a long-term follow-up study, so that it is more persuasive as evidence suggesting that reimplantation is unnecessary than other previous studies. There is a limitation in this study because it was a retrospective study, and the sample size is small. Prospective, randomized studies should be done to elucidate the need for simultaneous ureteral reimplantation with AC.

## Data Availability

The datasets used and or analyzed during the current study are available from the corresponding author on reasonable request.

## Abbreviations

AC: Augmentation cystoplasty
CIC: Clean intermittent catheterization
CKD: Chronic renal failure
DO: Detrusor overactivity
GFR: Estimated glomerular filtration rate
MACE: Malone’s antegrade continence enema
UTI: Urinary tract infection
UVJ: Ureterovesical junction
VUR: Vesicoureric reflux
